# Posterior Shoulder Instability but Not Anterior Shoulder Instability Is Related to Glenoid Version

**DOI:** 10.1016/j.asmr.2023.100794

**Published:** 2023-09-09

**Authors:** Thomas K. Moore, Conor J. Kilkenny, Eoghan T. Hurley, Bryan M. Magee, Jay M. Levin, Sami U. Khan, Jonathan F. Dickens, Hannan Mullett

**Affiliations:** a Sports Surgery Clinic, Dublin, Ireland; b Royal College of Surgeons in Ireland, Dublin, Ireland; c Duke University, Durham, North Carolina, U.S.A.

## Abstract

**Purpose:**

To assess and compare glenoid version in patients with anterior shoulder instability (ASI), posterior shoulder instability (PSI), and a control group.

**Methods:**

The operative notes of all patients that had undergone arthroscopic shoulder instability repair between January 2017 and May 2022 were retrospectively reviewed. Magnetic resonance imaging scans were then analyzed, and glenoid version was measured by a single blinded observer. A *P* value <.05 was considered statistically significant.

**Results:**

There were 100 patients included in the ASI group, 65 in PSI group, and 100 in the control group. The mean glenoid versions for the ASI group were −16°, −9.1°, and −9.2° for the vault version, simplified vault version, and chondrolabral version, respectively. The mean glenoid versions for the PSI group were −21°, −13.4°, and −16.6° for the vault version, simplified vault version, and chondrolabral version, respectively. The mean versions for the control group were −17.8°, −9.5°, and −9.8° for the vault version, simplified vault version and chondrolabral version, respectively. ANOVA testing and post hoc comparisons revealed the PSI group to be significantly more retroverted than both other groups *P* < .001. The ASI group’s degree of glenoid version was not significantly different to that of the control *P* = .009.

**Conclusion:**

Patients with PSI have a higher degree of retroversion in comparison to those with ASI and control. There is no significant difference in glenoid version among patients with ASI when compared with control.

**Level of Evidence:**

Level III, retrospective comparative study.

## Introduction

Shoulder instability is a very common pathology with the highest incidence rates occurring in males aged 16 to 20 years old.^1^ Glenoid version is the degree to which the glenoid fossa deviates, to face anteriorly or posteriorly, relative to the neutral position. Retroversion has been identified as the most significant risk factor for posterior shoulder instability (PSI), with an even higher rate of retroversion among those suffering atraumatic PSI, and as a risk factor for contralateral shoulder instability.^2^, ^3^, ^4^ Glenoid version can guide treatment for PSI, the posterior open wedge osteotomy may be indicated for patients with excessive retroversion and PSI, and has been suggested to be superior to Bankart repair when retroversion is >15°.^5^, ^6^, ^7^, ^8^

The literature describing the association between anterior shoulder instability (ASI) and glenoid version is scant in comparison to its posterior equivalent, and there is conflicting evidence reported. Hohmann et al. strongly suggested that glenoid version was significantly increased in ASI, and Aygün et al. concluded that glenoid version is an important factor in the development of ASI.^9^^,^^10^ In contrast, multiple studies have found that glenoid version is not a significant risk factor for ASI.^11^, ^12^, ^13^ Furthermore, glenoid version has been identified as a risk factor for postoperative recurrence following arthroscopic Bankart repair.^14^^,^^15^ Eichinger et al. showed that with increased glenoid anteversion, less force was required to cause an anterior dislocation of the shoulder.^16^ These studies are inconsistent in their methods, and thus, the true nature of the relationship between glenoid version and ASI is not known.

The purpose of this study was to assess and compare glenoid version in patients with anterior shoulder instability (ASI), posterior shoulder instability (PSI), and a control group. Our hypothesis was that the PSI group would show a higher degree of retroversion in comparison to both our control and ASI group and that our ASI group may show a lesser degree of retroversion in comparison to both our control and PSI group.

## Methods

### Data Collection

A retrospective review of all patients that had undergone an arthroscopic Bankart repair under a single fellowship trained shoulder surgeon between January 2017 and May 2022 was carried out. Inclusion criteria for the test groups were 1) unidirectional shoulder instability, either anterior or posterior, 2) managed with arthroscopic Bankart repair, 3) a preoperative MRA on the local hospital system. Anterior shoulder instability was defined as those that had more anterior sutures than posterior sutures on arthroscopic Bankart repair. Posterior instability was defined as those that had more posterior sutures than anterior sutures on arthroscopic Bankart repair. The control group had the following inclusion criteria: 1) patients who had undergone an arthroscopic rotator cuff repair under a single fellowship-trained shoulder surgeon and 2) patients had preoperative magnetic resonance imaging (MRI) on the local hospital system. Patients that met the criteria for the control group were sorted according to age at date of surgery, and the youngest 100 patients to meet the inclusion criteria were included in the control group. The medical notes of all patients included in the study were analyzed, and demographical data were gathered. Gender, age at date of surgery, and operative side were recorded. For the test groups, further data were gathered from the operative notes, including number of anterior sutures, number of posterior sutures, and grade of instability on examination under anesthetic.

**Fig 1:**
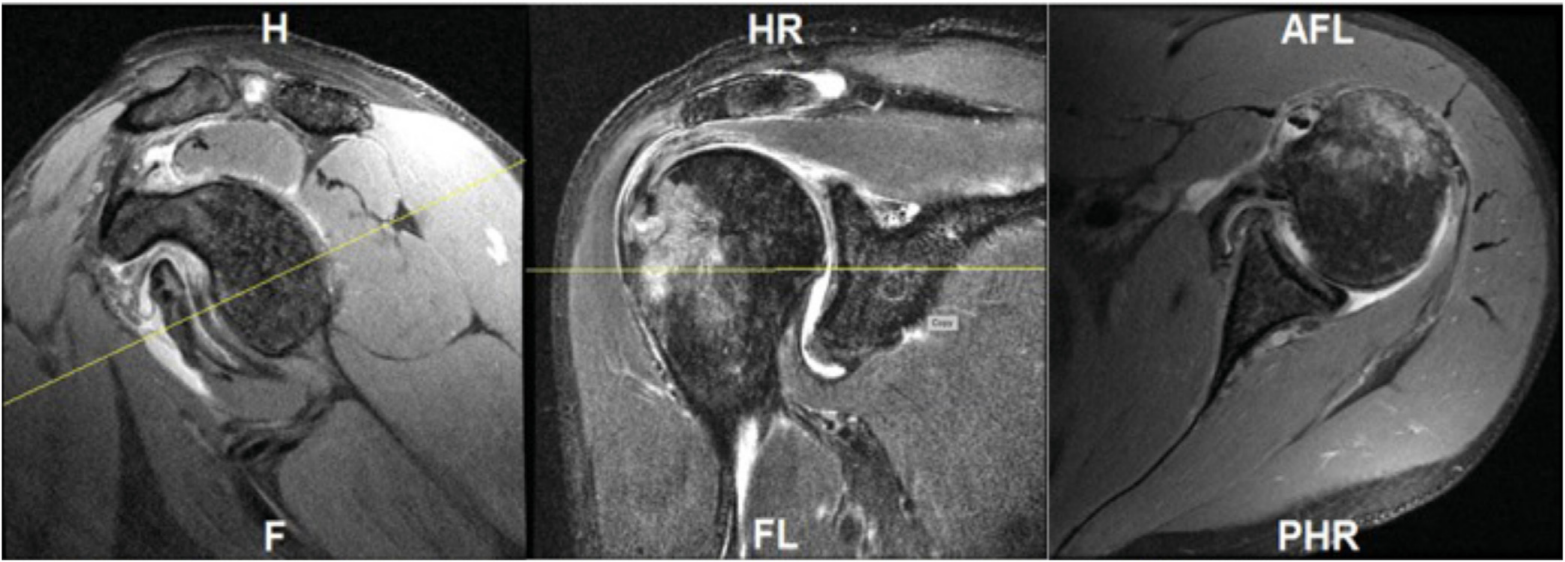
Sagittal, coronal, and transverse images were used to select the transverse image most central on the glenoid.

All patients received preoperative 3-Tesla MRA (MRI in the control group) scans (TwinSpeed 8; GE Medical Systems). The shoulder was in a neutral position and placed on a dedicated shoulder surface coil. Coronal, sagittal, and axial T1-weighted, fat-saturated images as well as T2-weighted, fat-saturated coronal images were obtained. They were analyzed by a single blinded observer. First, the glenohumeral joint was viewed from coronal, sagittal and transverse planes and the midpoint of the glenoid face was identified (Fig 1). The transverse slice in closest proximity to the midpoint was selected. This image was then used for all methods.

#### Simplified Vault Method (Matsumura)

**Fig 2:**
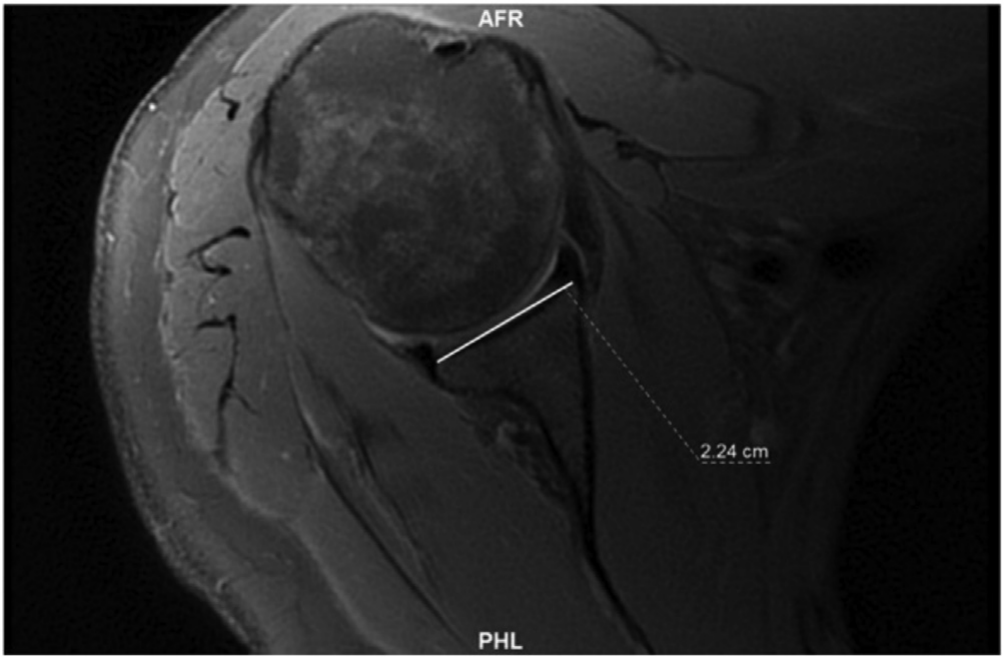
A line was drawn between the anterior and posterior osseus glenoid rims (glenoid line).

**Fig 3:**
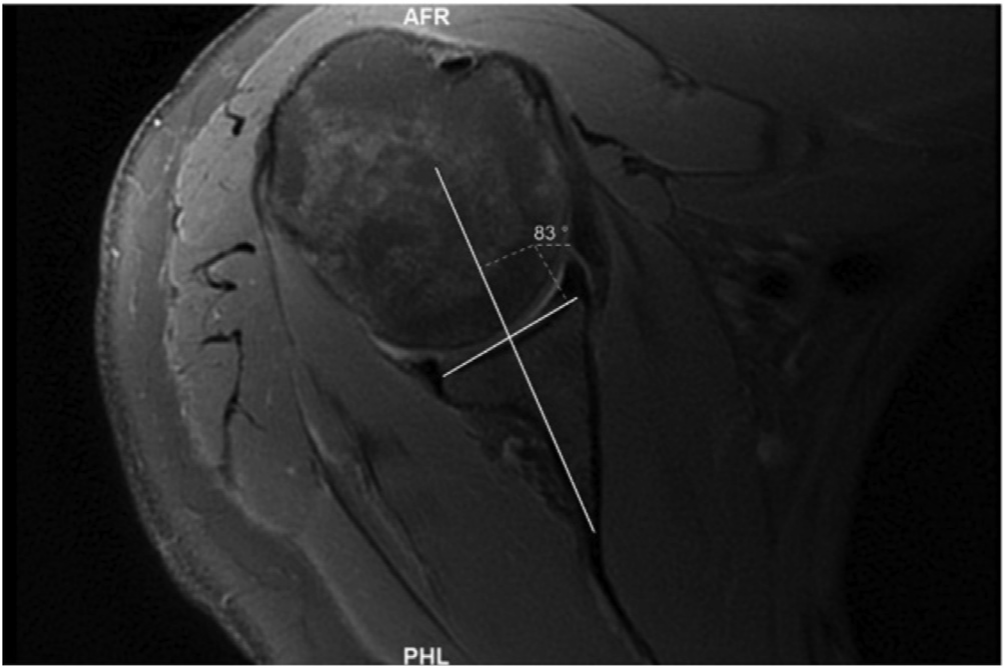
A line was then drawn from the medial corner of the triangle created by the glenoid vault through a point that bisected the glenoid line (simplified vault line).

A line was drawn between the anterior and posterior osseus glenoid rims (glenoid line) (Fig 2). A line was then drawn from the medial corner of the triangle created by the glenoid vault through a point that bisected the glenoid line (simplified vault line) (Fig 3). The angle between these lines was subtracted from 90, and this gave us the angle of osseus glenoid version. This was described as simplified vault version.^17^ A positive number indicated retroversion, and this was recorded as a negative number to reflect retroversion. A negative number indicated anteversion and was recorded as a positive number to reflect anteversion.

#### Chondrolabral Method (Maister)

**Fig 4:**
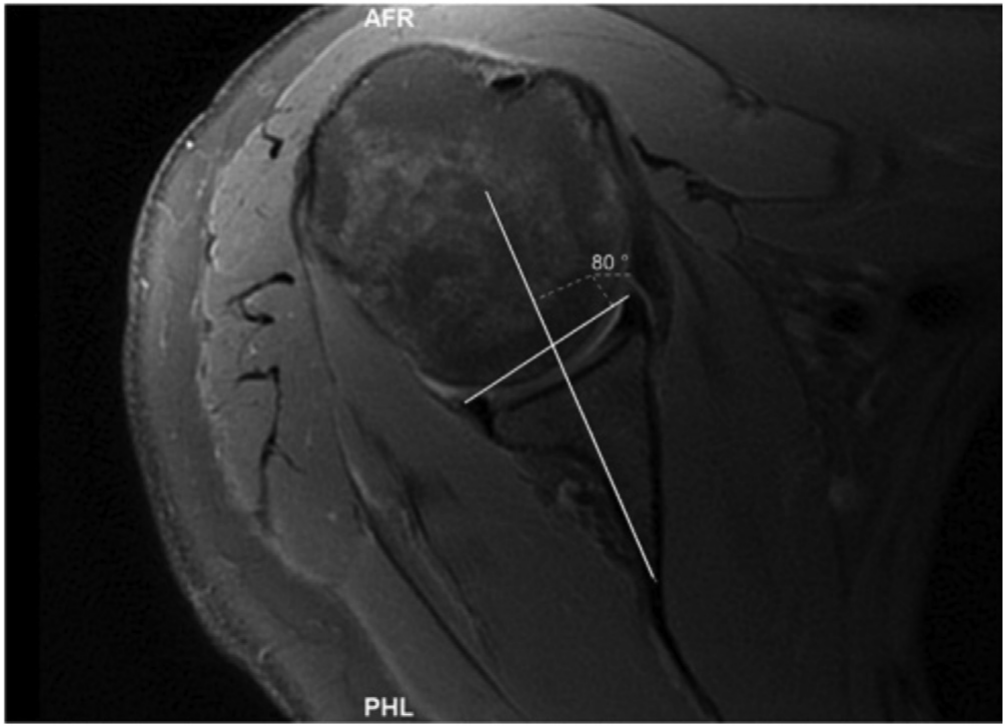
A line drawn between the anterior and posterior chondrolabral rims on the same slice already used. The angle between this line and the simplified vault line was subtracted from 90 in order to give us the chondrolabral version.

The image selected was approximately at the midpoint of the glenoid face and was thus equivalent to the “50% point” described by Maister et al. This method has two components. The first being identical to the simplified vault method.^18^ The second component of this method involves a line drawn between the anterior and posterior chondrolabral rims on the same slice already in use (chondrolabral line) (Fig 4). The angle between this line and the simplified vault line was subtracted from 90 in order to give us the chondrolabral version. A positive number indicated retroversion, and this was recorded as a negative number to reflect retroversion. A negative number indicated anteversion and was recorded as a positive number to reflect anteversion. We did not measure the angle at 25% or 75% of the long axis of the glenoid on viewing in the coronal plane.

#### Vault Method (Poon-Ting)

**Fig 5:**
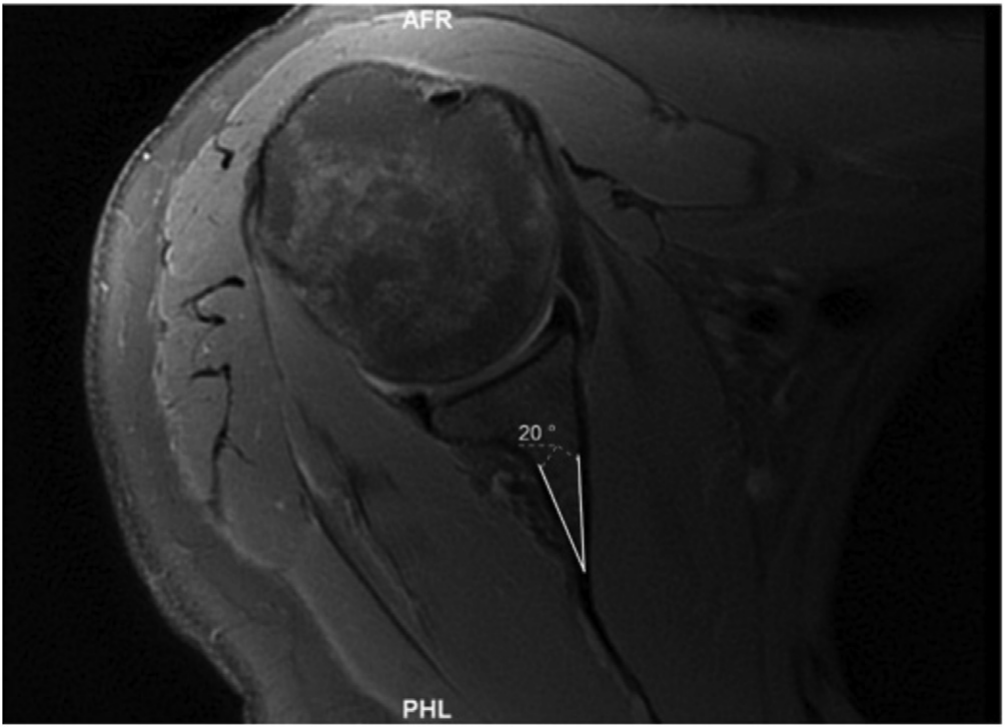
An isosceles triangle is created in the glenoid vault.

**Fig 6:**
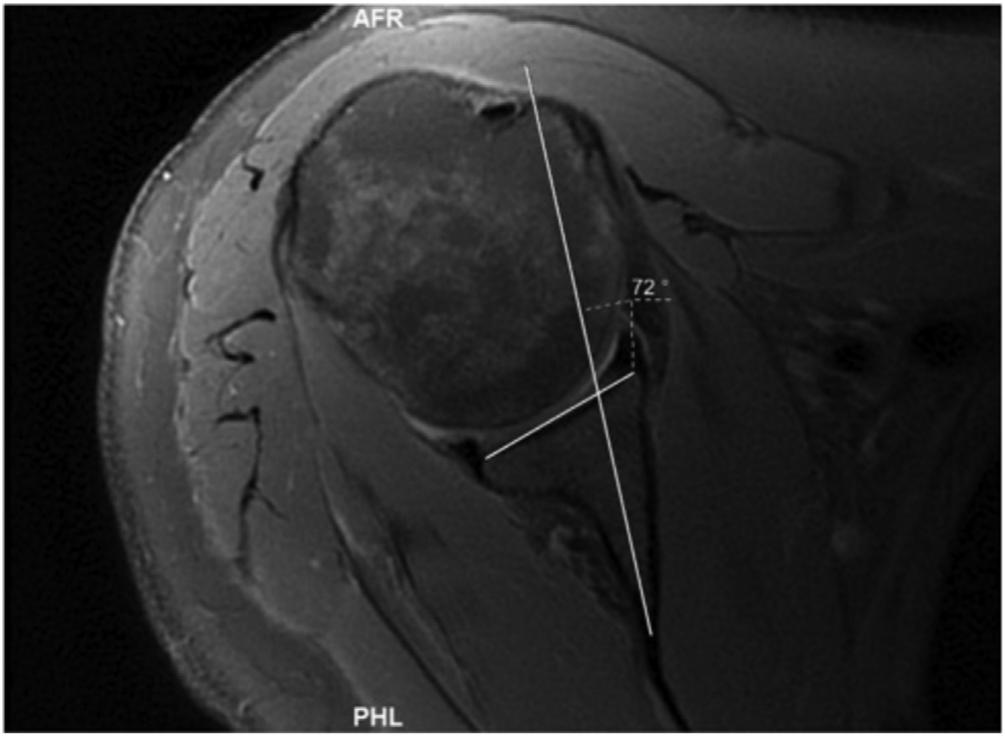
A line was then drawn from the apex of the isosceles triangle that bisected the angle of this vertex through the glenoid line (Poon-Ting Line). The angle between the Poon-Ting line and the glenoid line was subtracted from 90 to give the angle of glenoid.

This was calculated by first creating an isosceles triangle in the glenoid vault (Fig 5).^19^ A line was then drawn from the apex of the isosceles triangle that bisected the angle of this vertex through the glenoid line (Poon-Ting line) (Fig 6). The angle between the Poon-Ting line and the glenoid line was subtracted from 90 to give the angle of vault version. A positive number indicated retroversion, and this was recorded as a negative number to reflect retroversion. A negative number indicated anteversion and was recorded as a positive number to reflect anteversion.

### Statistical Analysis

Quantitative statistical analysis was performed. Demographic information such as gender, age at date of surgery and operative side analyzed according to their direction of instability. Box plots were created to visually represent the data. ANOVA testing and post hoc comparisons were carried out to analyze the differences between groups. This was done using the jamovi project (2021). (version 2.2) [computer software; retrieved from https://www.jamovi.org]. An intraobserver reliability calculation was not performed. A *P* value < .05 was considered statistically significant.

## Results

### Patient Demographics

Overall, 581 arthroscopic shoulder stabilizations had been performed by a single shoulder surgeon in the time period. 100 shoulders met the inclusion criteria for the ASI group. Sixty-five shoulders met the inclusion criteria for the PSI group. The control group was formed as described and included 100 patients. The mean age in years for each group was 25.9 for the ASI group, 24.8 for the PSI group and 40.6 for the control. 83/100 (83%) of the ASI group were men, 62/65 (95.4%) were men in the PSI Group, 84/100 (84%) in the control. There were 39 left and 61 right shoulders for the ASI group, 20 right and 45 left shoulders in the PSI group, and 48 left and 52 right shoulders in the control group.

### Glenoid Version

**Fig 7:**
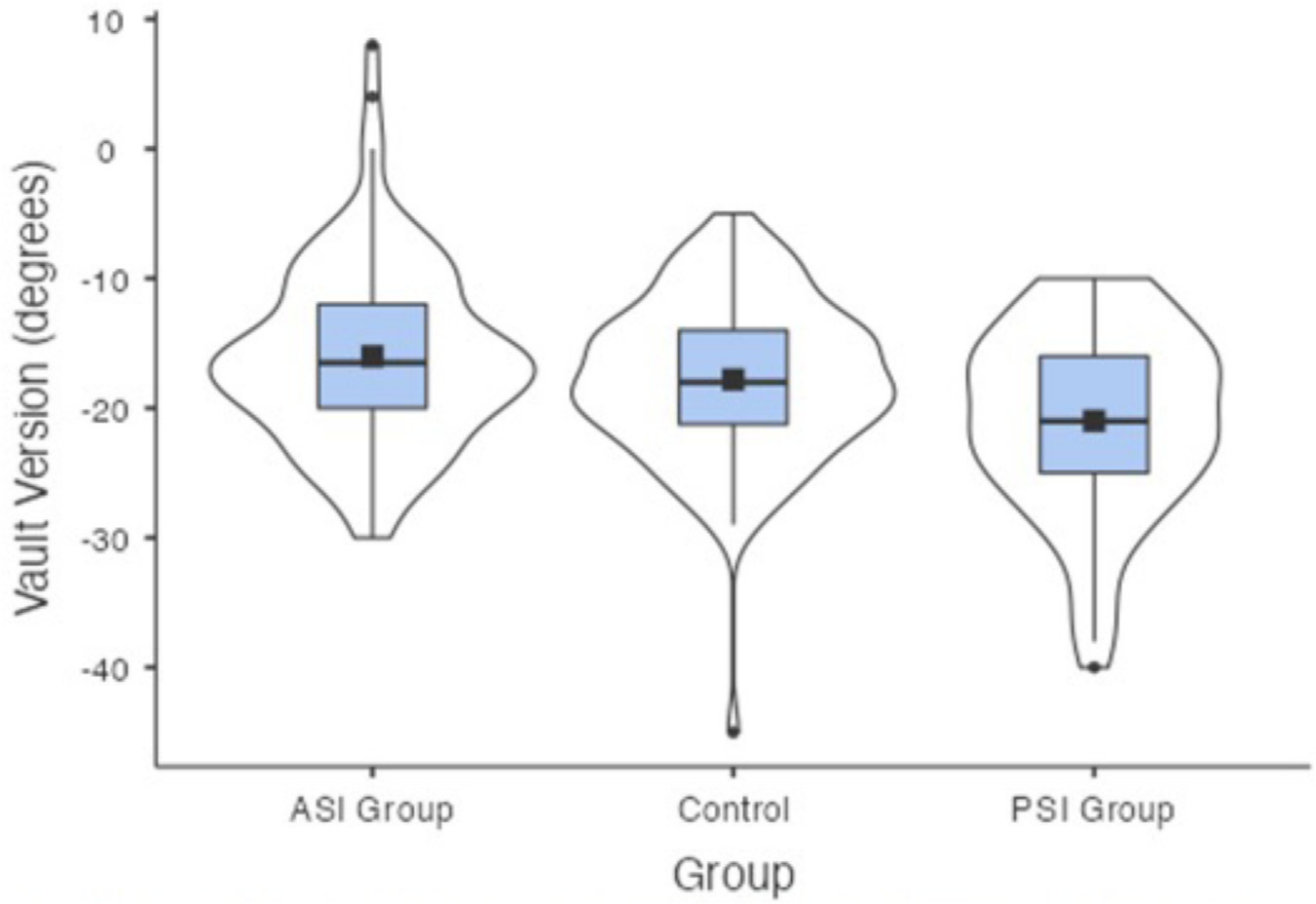
Box plot presenting the vault version for each group, the solid black box represents the mean, the black line represents the median, the blue box represents the middle 50% of the data, and the violin represents the distribution.

**Fig 8:**
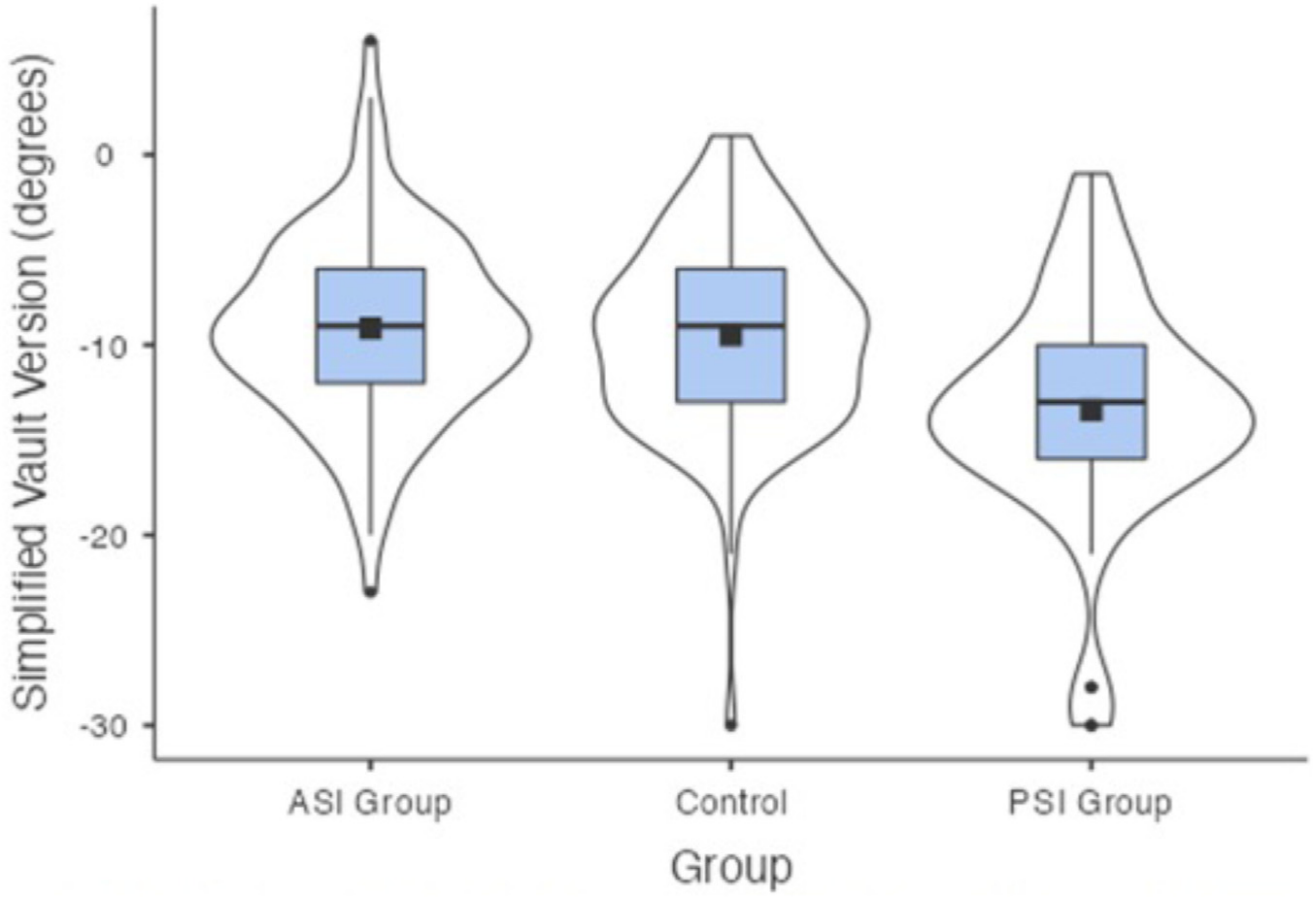
Box plot presenting the simplified vault version for each group, the solid black box represents the mean, the black line represents the median, the blue box represents the middle 50% of the data, and the violin represents the distribution.

**Fig 9:**
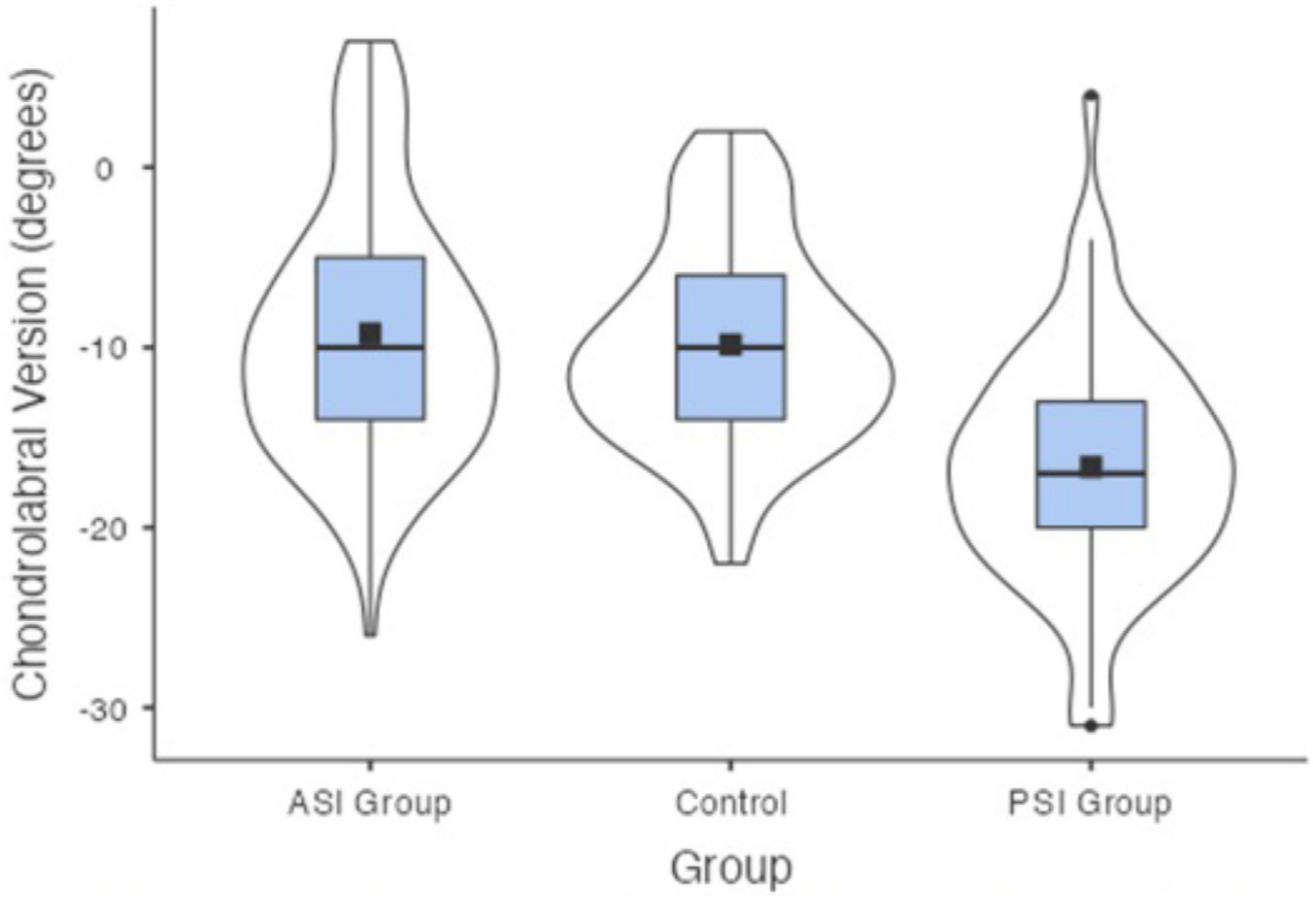
Box plot presenting the chondrolabral version for each group, the solid black box represents the mean, the black line represents the median, the blue box represents the middle 50% of the data, and the violin represents the distribution.

The mean glenoid versions for the ASI group were −16°(SD = 7°, CI = −14.6° to −17.4°), −9.1° (SD = 4.9°, CI = −8.2° to −10.1°), −9.2° (SD = 7°, CI = −7.8° to −10.6°) for the vault version, simplified vault version and chondrolabral version respectively. The mean glenoid versions for the PSI group were −21° (SD = 7.1°, CI = −19.3° to −22.7°), −13.4° (SD = 5.9°, CI = −12° to −14.9°), −16.6° (SD = 6.4°, CI = −15.1° to −18.2°) for the vault version, simplified vault version and chondrolabral version respectively. The mean versions for the control group were −17.8° (SD = 6.2°, CI = −16.6° to −19°), −9.5° (SD = 4.9°, CI = −8.5° to −10.4°), −9.8° (SD = 5.5°, CI = −8.8° to −10.9°) for the vault version, simplified vault version and chondrolabral version, respectively (Table 1, Figs 7, 8, and 9).

**Table 1:** Results by Group

	Group	*N*	Mean	95% Confidence Interval		SD
				Lower	Upper	
Age (years)	ASI Group	100	25.90	24.2	27.61	8.74
	Control	100	40.61	39.0	42.26	8.40
	PSI Group	65	24.83	23.2	26.41	6.50
Vault version (°)	ASI Group	100	−16.01	−17.4	−14.64	6.97
	Control	100	−17.80	−19.0	−16.59	6.17
	PSI Group	65	−20.98	−22.7	−19.26	7.11
Simplified vault version (°)	ASI Group	100	−9.12	−10.1	−8.16	4.88
	Control	100	−9.48	−10.4	−8.51	4.93
	PSI Group	65	−13.43	−14.9	−12.00	5.88
Chondrolabral version (°)	ASI Group	100	−9.22	−10.6	−7.84	7.02
	Control	100	−9.84	−10.9	−8.76	5.52
	PSI Group	65	−16.63	−18.2	−15.06	6.44

ASI, anterior shoulder instability; PSI, posterior shoulder instability.

### Statistical Analysis

The PSI group was significantly more retroverted than the ASI group by a mean of 7.4°, 4.3° and 5° for the vault version, simplified vault version, and chondrolabral version, respectively. The PSI group was significantly more retroverted than the control group by a mean of 6.8°, 4°, and 3.2° for the vault version, simplified vault version and chondrolabral version respectively. The *P* values were <.001 for all differences except when comparing the vault version between control and PSI groups, where *P* values were .009 and .01 for Tukey and Bonferroni corrections, respectively. ASI and control groups showed no significant differences in their means (Table 2).

**Table 2:** Post Hoc Comparisons

Post Hoc Comparisons - Vault Version							
Comparison							
Group	Group	Mean Difference (°)	SE	df	*t*	*P*_Tukey_	*P*_Bonferroni_
ASI Group	Control	1.79	0.949	262	1.89	.145	.182
	PSI Group	4.97	1.070	262	4.65	<.001	<.001
Control	PSI Group	3.18	1.070	262	2.98	.009	.010

Post Hoc Comparisons - Simplified Vault Version							
Comparison							
Group	Group	Mean Difference (°)	SE	df	*t*	*P*_Tukey_	*P*_Bonferroni_
ASI Group	Control	0.360	0.730	262	0.493	.875	1.000
	PSI Group	4.311	0.822	262	5.242	<.001	<.001
Control	PSI Group	3.951	0.822	262	4.804	<.001	<.001

Post Hoc Comparisons - Chondrolabral Version							
Comparison							
Group	Group	Mean Difference (°)	SE	Df	*t*	*P*_Tukey_	*P*_Bonferroni_
ASI Group	Control	0.620	0.898	262	0.691	.769	1.000
	PSI Group	7.411	1.011	262	7.328	< .001	< .001
Control	PSI Group	6.791	1.011	262	6.715	<.001	<.001

## Discussion

The most important finding of this study was that the PSI group was significantly more retroverted than the control and ASI groups. This is in keeping with prior literature.^3^^,^^11^^,^^20^ Interestingly, there was no significant difference between the mean of the control group and the mean of the ASI group. Our findings suggest an individual with ASI may have a similar degree of glenoid version as that of an individual with no shoulder instability. The literature describing the association between ASI and glenoid version is scant in comparison to its posterior equivalent, and there is conflicting evidence reported. Our findings are in contrast with the findings of Hohmann et al., who found that glenoid retroversion was significantly less in those with ASI.^9^ Similar to our study, MRI was used by Hohmann et al. to compare an ASI group (*n* = 128) of young patients with a control group (*n* = 130) of older patients; however, Hohmann et al. measured the glenoid version using a method described by Tetreault et al.^21^ Aygün et al. found in their study group that the glenoid version angles on the dislocated side were significantly more anteverted compared to shoulders in the control group and concluded that glenoid version “is an important factor in the development of anterior dislocation of the shoulder”.^10^ However, when comparing our findings with theirs, it is important to consider the numerous differences between the studies. They had fewer patients enrolled, had an older cohort in their study group, and used the Friedman method applied to computed tomography (CT) to measure the version.

Similar to our results, Mehl et al. reported significantly higher levels of retroversion among their PSI group in comparison to their ASI group when using the vault method applied to MRI.^22^ Despite having similar values for retroversion in their ASI and PSI groups, comparison to our study is limited as Mehl et al.’s study lacked a control group. Privitera et al. conducted a similar study to ours in 2016.^11^ They used MRI to compare ASI, PSI, and control groups with both the vault and Friedman method. They found a significantly higher degree of retroversion in the PSI group when using the Friedman method. However, Privitera et al.’s groups were small in comparison to ours with 30-34 patients per group. Using a CT-based method of measurement, Peltz et al. compared multiple aspects of glenohumeral morphology, including version and found no significant difference between patients with ASI and a control.^12^ Our results are in keeping with much of the literature, despite the inconsistencies surrounding the relationship between ASI and glenoid version.

In order to say whether a pathological shoulder is anteverted or retroverted, one must establish a normal value for glenoid version. Our control group represented normal glenoid version for each method; thus, we can say normal glenoid version is approximately −17.8° (SD = 6.2°, CI = −16.6° to −19°), −9.5° (SD = 4.9°, CI = −8.5° to −10.4°), −9.8°(SD = 5.5°, CI = −8.8° to −10.9°) for vault version, simplified vault version, and chondrolabral version, respectively. This is similar to Poon et al.’s findings of their control group, which was a mean of −19° (±3°) when using the vault method, Matsumura et al.’s result of −8.9° (±2.7°) when using the simplified vault method and Maister et al.’s chondrolabral version results of approximately −10°.^17^, ^18^, ^19^ The results of our control group were similar to the results of other articles using these methods. This suggests that these methods are reproducible and consistent.

We found that all 3 methods used were in agreement that the PSI group was significantly more retroverted than the ASI and control groups. Privitera et al.’s results showed a lack of consistency between different methods. When using the Friedman method, they found a significant difference in the glenoid version when comparing the PSI group with the ASI and control groups, but when using the vault method, they did not find a significant difference between any group.^11^ This difference is likely caused by methods using various reference points from which to establish neutral version and, thus, define glenoid version slightly differently.

Regarding the Friedman method, concern has been raised over the use of the medial scapular border as a reference point, as it is subject to a high level of anatomical variance, and the entire body of the scapula is often not visible on standard MRA of the shoulder.^19^^,^^23^^,^^24^ Poon et al. devised the vault method in an effort to reduce the anatomical variation affecting the measurement of the glenoid version. This method uses the endosteal vault to generate a line of neutrality and has become popular in recent years. The Poon-Ting method has been compared with the Friedman method and is considered a valid method; however, the Poon-Ting method was shown to have lower levels of interobserver and intraobserver reliability.^25^

Although the literature describing the association between ASI and glenoid version is unclear, there is evidence that anteversion of the glenoid may be a risk factor for recurrence of instability following arthroscopic Bankart repair. Cheng et al. found that patients with retroversion less than 6° were 9.1 times more likely to have recurrent instability.^14^ They suggested that this may be due to the obvious mechanical susceptibility of an anteverted glenoid to anterior instability. However, they also discussed the possibility that the anteversion observed in post-Bankart patients could be a result of glenoid bone loss rather than inherent anteversion. Subsequently, Li et al. demonstrated that glenoid anteversion was not a risk factor for recurrence in individuals without glenoid bone loss but was a risk factor in those with subcritical glenoid bone loss.^15^ Therefore, it remains uncertain whether glenoid anteversion is a risk factor for ASI or a consequence thereof.

The current literature surrounding the association between glenoid version and instability is heterogenous. The lack of a standardized method of measurement for glenoid version leads to a lack of coherence among the literature and impedes cross comparison. The author of this article could find more than 10 methods of measuring glenoid version among the literature. The definition of glenoid version varies between articles. Our use of 3 different methods gives us a broader idea of the true level of glenoid version present in a scapula. However, all methods in our study generate a line of neutral version from points within the endosteal vault, this limits our ability to compare these results with studies that use other methods of measurement. Many studies use more than one observer in the measurement of the glenoid version angle; however, given it has been shown that intraobserver reliability is higher than interobserver reliability in the measurement of glenoid version with MRI, the authors felt that a single observer did not diminish the results of the study.^26^

The inclusion of patients with rotator cuff pathology in our control group raises questions about the association between rotator cuff pathology and glenoid version. Although some studies have found an association, others have shown no significant link.^21^^,^^27^^,^^28^ The mean age of our control is greater than that of the study groups by ∼15 years. One could raise concern over age-related osteoarthritic change affecting the degree of version; however, this is typically observed in older age groups.^29^ It has been suggested that with increased age, the glenoid becomes more anteverted, which potentially masks an underlying increased level of anteversion among our ASI group.^30^ These factors should be considered when interpreting the results.

### Limitations

This study is not without limitations. One is the retrospective nature of the study. Another is that patients were not screened to assess for additional shoulder pathology. We did not assess functional outcome measures, and, thus, we could not assess the impact of glenoid version on outcomes following arthroscopic Bankart repair. We could not identify whether there were a critical level of version that would lead to increased risk of recurrence. We were unable to use the Friedman method, and we, thus, cannot compare it with the methods used.

## Conclusions

Patients with PSI have a higher degree of retroversion in comparison to those with ASI and control. There is no significant difference in glenoid version among patients with ASI when compared with control.

## Supplementary Data

ICMJE author disclosure forms
